# Evaluation of the Clinical Performance of NuSmile Pedodontics Zirconia Crowns in Pulp-Treated Primary Teeth—2 Years Follow-Up Study

**DOI:** 10.1055/s-0041-1742129

**Published:** 2022-02-23

**Authors:** Rana A. Alamoudi, Tarun Walia, Dina Debaybo

**Affiliations:** 1 Department of Pediatric Dentistry, Faculty of Dentistry, King Abdulaziz University, Jeddah, Saudi Arabia; 2 Department of Clinical Sciences, College of Dentistry, Ajman University, United Arab Emirates; 3 Dr. Dina's Pediatric Dentistry Clinic, Dubai, United Arab Emirates

**Keywords:** Zirconia pediatric crowns, NuSmile, clinical success, primary teeth, incisors, molars

## Abstract

**Objectives** There is a lack of information regarding the longevity of prefabricated crowns on primary teeth. Therefore, the objective of the present study was to evaluate the clinical success rate of NuSmile pediatric Zirconia crowns in both primary anterior and posterior teeth up to 2 years in terms of gingival health, levels of plaque, restoration failure, and marginal integrity.

**Materials and Methods** This interventional study evaluated the clinical performance of NuSmile primary Zirconia crowns on 232 (172 primary incisors and 60 primary molars) pulp-treated primary teeth. Statistical analysis included independent *t* -test and Kaplan–Meier survival method for which the level of significance was set up at *p* < 0.05.

**Results** Gingival and plaque index of the teeth restored with NuSmile Zirconia crowns (test tooth) compared with adjacent and antagonist teeth (control tooth) showed no statistically significant difference at all time intervals ( *p* > 0.05). The Kaplan–Meier survival graph indicated that only 34% of NuSmile Zirconia incisor crowns and 86% of NuSmile Zirconia molar crowns have survived at the end of 24 months. Of the 172 NuSmile Zirconia incisor crowns placed, only 82 teeth survived at the end of 2 years and the majority of the crowns completely lost the Zirconia crown. The Kaplan–Meier graph also indicated that more than two-thirds of crowns, i.e., 90% incisor crowns and 76% of molar crowns, had good marginal integrity at the end of 24 months.

**Conclusions** Zirconia pediatric crowns preserve and maintain gingival health and have long-term survival rates with good retention and marginal integrity, indirectly preventing secondary caries. Hence, Zirconia pediatric crowns are a well-suited restoration of choice for primary teeth rehabilitation.

## Introduction

In today's modern society, both parents and children are cosmetically conscious and increasingly concerned about the esthetics of their teeth. The child's parents often influence the dental professionals treating the children in selecting dental restoration, and parents are getting more involved in clinical decision-making than ever before. 1 A recent study has also shown that children at the age of 6 years and above can appreciate the esthetics of their anterior teeth. 2 Thus, there is a great preference for restorations that bring the primary tooth back to a healthy state in both appearance and function.

Management of extensive carious lesions and traumatized primary teeth has gradually shifted from extraction to full-coverage restorations. Extra-coronal full-coverage crowns are indicated in primary teeth with developmental defects, multi-surface caries, patients with high caries risk, fractured teeth, teeth where direct restoration tends to fail, after pulpal therapy, as abutments for space maintainers, and teeth with excessive wear. 3 4

Traditionally, composite strip crowns and stainless steel crowns (SSC) have been used as full-coverage restorations for grossly decayed and pulp-treated primary anterior teeth and molars. Strip crowns are esthetically better but are technique sensitive. Moisture and hemorrhage control is essential with strip crowns as it could lead to resin placement failure. SSC crowns have been successful for many years in terms of durability, retention, and function. The most significant problem was their poor esthetics limiting their use to the posterior segment only. 5 However, some parents refuse SSC due to their black color, which is unappealing.

Pre-veneered stainless steel crowns (PSSC) combine conventional SSC's durability with the esthetic appeal of a composite resin veneer. However, the significant concerns with PSSC were removing additional coronal tooth structure, inability to crimp the margins of the crowns before cementation, and loss of the esthetic acrylic facing. 3 6

Prefabricated pediatric Zirconia crowns offer an excellent alternative to full-coverage crowns when restoring deciduous teeth with a sizeable carious defect. These crowns have high flexural strength, allowing them to resist crack propagation. The additional benefits of these crowns are the ability to replace metals due to extremely high strength and toughness, higher resistance to chemicals, and superior erosion resistance. 7 Zirconia crowns are biocompatible, autoclavable, and equal to or more durable than natural enamel.

Since the introduction of primary Zirconia crowns, several studies have reported their clinical success. Severely mutilated primary anterior teeth showed that NuSmile Zirconia crowns (NuSmile Ltd., Houston, TX, United States) offered superior esthetics and durability with remarkable gingival responses up to 24 months. 8 A clinical study on the wear behavior of primary enamel against Zirconia crowns demonstrated the lowest wear rate of primary enamel. 9 A retrospective study that evaluated the clinical success and parental satisfaction showed a considerable percentage (89%) of parents were highly satisfied by the crowns' size, color, and form. 10 Additionally, a study on three randomized controlled anterior aesthetic full-coverage crowns showed Zirconia crowns to be highly retentive compared with resin composite strip crowns and PSSC. 1 To date, most clinical studies of prefabricated crowns have been conducted on anterior teeth. There is a lack of information regarding the longevity of such crowns on primary teeth. Therefore, the objective of the present study was to evaluate the clinical success rate of NuSmile pediatric Zirconia crowns in both primary anterior and posterior teeth up to 2 years in terms of gingival health, plaque levels, restoration failure, and marginal integrity.

## Materials and Methods

### Study Protocol

This interventional study was done between 2014 and 2017 in accordance with the Declaration of Helsinki (as revised in Edinburgh 2000). It evaluated the clinical performance of NuSmile primary Zirconia crowns on 75 children (40 males and 35 females) aged 2 to 8 years. The study included healthy and cooperative children managed by non-pharmacological and pharmacological behavioral management techniques. Children with no history of systemic illness and parents who signed the consent on behalf of their children and were willing to follow-up during the entire course of the study formed the study cohort. Inclusion criteria for the study were primary teeth with sufficient tooth structure and expected to survive for at least 2 years. Grossly carious non-restorable clinical crown, primary tooth with root resorption, tooth exfoliated within 1 year of crown placement, special need children, and parents not willing to come for follow-up were excluded from the study.

Pulpal status was assessed clinically and confirmed by pre-operative periapical radiographs. Around 232 (172 primary incisors and 60 primary molars) pulp-treated primary teeth received a prefabricated Zirconia crown ( Table 1 ). The study cohort consisted of 52 pulpotomy and 180 pulpectomy cases. Two clinicians (T.W. and D.D.) with experience of more than 20 years of clinical practice performed pulpal treatment and crown placement in the same appointment. Teeth with non-vital pulp that required two visit pulpectomy were done, and the crowns were cemented on the day of root canal obturation. Endodontic procedures for pulpally involved teeth were performed as per the best policy clinical practice guidelines from the American Academy of Pediatric Dentistry Reference Manual 2020. 11

**Table 1:** Distribution of NuSmile Zirconia crowns as per the type of teeth

Primary teeth	Maxillary teeth	Mandibular teeth	Pulpotomy	Pulpectomy
Central incisors	104	–	–	104
Lateral incisors	68	–	–	68
First primary molars	8	17	22	3
Second primary molars	5	30	30	5

The same operator who placed the crown recorded clinical parameters such as gingival index, plaque index, restoration failure, and marginal integrity at 6, 12, 18, and 24 months. Both operators were standardized to evaluate index scores. The gingival index was recorded using a blunt periodontal probe, and a plaque disclosing swab was used to assess the plaque index. The gingival health and plaque scores were evaluated and compared with adjacent and opposing teeth. The evaluation of restoration failure and marginal integrity was done clinically with visual assessment of restoration, according to the United States Public Health Service criteria ( Table 2 ).

**Table 2:** Evaluation criteria for gingival health, plaque index, restorative failure, and marginal integrity of NuSmile Zirconia crown

Criteria	Grades	Description	Author
Gingival health	0	No obvious signs of inflammation	Löe and Silness gingival index
	1	Mild marginal gingivitis tissue slightly reddened and edematous	
	2	Moderate marginal gingivitis tissue obviously reddened and edematous	
	3	Severe gingivitis tissue is very swollen: spontaneous bleeding	
Plaque index	0	No plaque	The United States Public Health Service
	1	Film at the gingival margin	
	2	Moderate accumulation	
	3	Abundance of plaque	
Restoration failure	0	Crown appears normal: no cracks, chips, or fractures	The United States Public Health Service
	1	Small but noticeable area of loss of material	
	2	Complete loss of the crown	
	E	Exfoliated	
Marginal integrity	0	No detectable margin	The United States Public Health Service
	1	Detectable margin	
	2	Infra-occlusion	
	E	Exfoliated	

### Clinical Procedure for NuSmile Zirconia Crown Preparation

Both operators prepared pulp-treated teeth for NuSmile Zirconia crowns as per the manufacturer's guidelines on crown placement protocol. There was no formal training or standardization between the two operators for crown placements except following technical guide instructions mentioned and recommended for use and general information on NuSmile Zirconia crowns. Before the tooth preparation, the operator chose a well-fitting prefabricated crown based on the pulp-treated tooth's original size. Local anesthesia was given using lidocaine 2% with epinephrine (1:80,000), especially when placing crowns without general anesthesia. Tooth structure of 1–1.5 mm was removed from the natural occlusal contours followed by 2 mm sub-gingival reduction with a special crown cutting kit provided by NuSmile. This opened up the interproximal contact areas and reduced the entire clinical crown by 20% (or 0.5–1.25 mm). All line angles of the prepared tooth were rounded. The crown's fit was checked, and crowns were cemented with Ketac Cem luting cement. Excess cement was removed, and crowns were held in place until the cement had been set.

## Results

Data were analyzed for descriptive data by SPSS version 20.0. Inferential statistics to compare gingival health scores/plaque level scores over the period between test tooth (NuSmile Zirconia crowns) and control tooth (adjacent/ antagonist tooth) was done using independent samples *t* -test. The Kaplan–Meier survival method was used to analyze restoration failure and marginal integrity of Zirconia crown for 2 years. The 95% confidence interval (CI) was included for stating the relevance of the finding, and the level of significance was set up at *p* < 0.05. The exfoliated teeth restored with NuSmile Zirconia crowns were evaluated for restoration failure and marginal integrity.

### Gingival Health and Plaque Scores

**Fig. 1:**
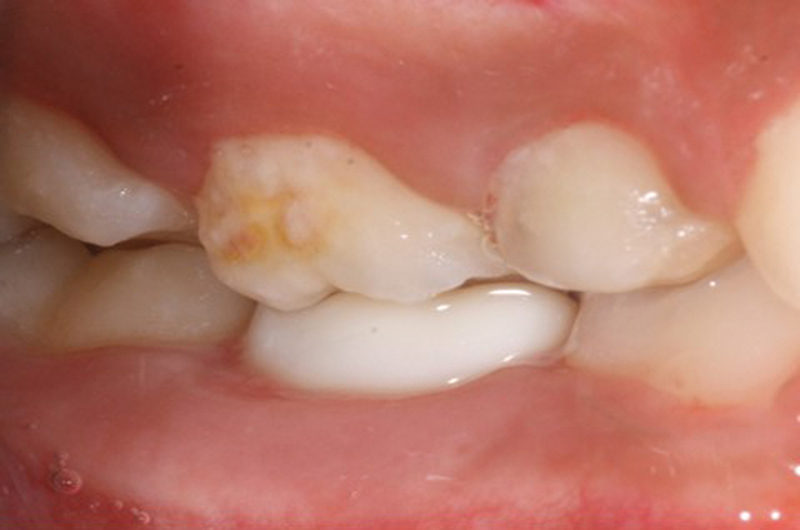
Deposits of plaque on maxillary second molar compared with prefabricated Zirconia mandibular second crown.

**Fig. 2:**
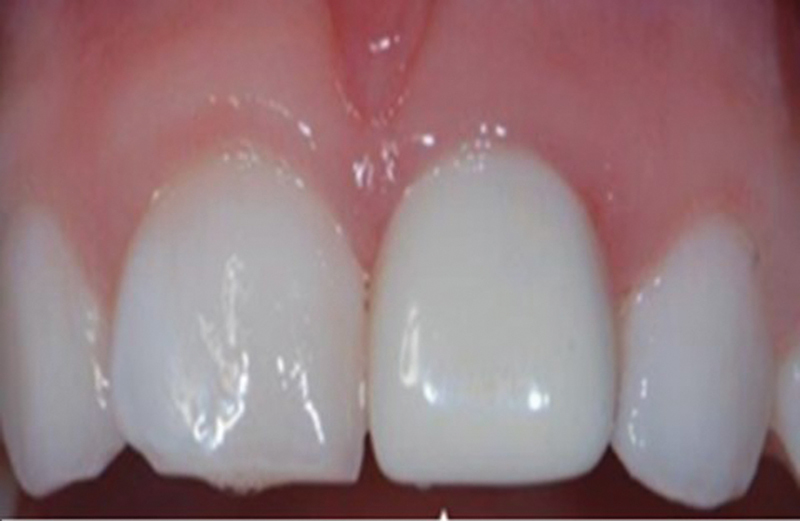
Deposits of plaque on right deciduous incisor compared with prefabricated Zirconia crown on the left incisor.

Tables 3 and 4 show the mean gingival health and plaque scores of normal and prefabricated Zirconia crowns at intervals up to 24 months. The oral health domain of clinical performance determined by the gingival and plaque index of teeth restored with NuSmile Zirconia crowns (test tooth) compared with adjacent and antagonist teeth (control tooth) had no statistically significant difference at all time intervals ( *p* > 0.05). Fig. 1 shows plaque deposits on maxillary second molar compared with prefabricated Zirconia mandibular second crown, while Fig. 2 shows plaque deposits on right deciduous incisor compared with prefabricated Zirconia crown on the left incisor.

**Table 3:** Comparison of gingival health scores of normal and prefabricated Zirconia crowns at 6-, 12-, 18-, and 24 months intervals

	Incisor						Molar					
Duration	Normal crown		Zirconia crown		*t*	*p* -Value	Normal crown		Zirconia crown		*t*	*p* -Value
	Mean	SD	Mean	SD			Mean	SD	Mean	SD		
6 months	1.45	0.55	1.35	0.45	329	1.96 ^ns^	1.99	0.45	1.35	0.50	117	1.96 ^ns^
12 months	1.50	0.55	1.35	0.50	319	1.96 ^ns^	2.05	0.50	1.50	0.60	104	1.98 ^ns^
18 months	1.50	0.45	1.40	0.50	290	1.96 ^ns^	2.10	0.65	1.60	0.45	101	1.98 ^ns^
24 months	1.55	0.55	1.35	0.60	293	1.96 ^ns^	2.30	0.45	1.70	0.50	104	1.98 ^ns^

Abbreviations: ns, not significant; SD, standard deviation.

**Table 4:** Comparison of plaque scores of normal and prefabricated Zirconia crowns at 6-, 12-, 18-, and 24 months intervals

Duration	Incisor						Molar					
Duration	Normal crown		Zirconia crown		*t*	*p* -Value	Normal crown		Zirconia crown		*t*	*p* -Value
	Mean	SD	Mean	SD			Mean	SD	Mean	SD		
6 months	1.05	0.60	0.89	0.45	329	1.96 ^ns^	1.45	0.60	0.96	0.45	110	1.96 ^ns^
12 months	1.20	0.60	0.95	0.55	322	1.96 ^ns^	1.65	0.65	1.05	0.50	111	1.98 ^ns^
18 months	1.20	0.60	1.00	0.40	270	1.96 ^ns^	2.15	0.55	1.20	0.50	113	1.98 ^ns^
24 months	1.20	0.60	0.95	0.45	273	1.96 ^ns^	2.15	0.55	1.20	0.50	103	1.98 ^ns^

Abbreviations: ns: not significant; SD: standard deviation.

### Restoration Failure

**Fig. 3:**
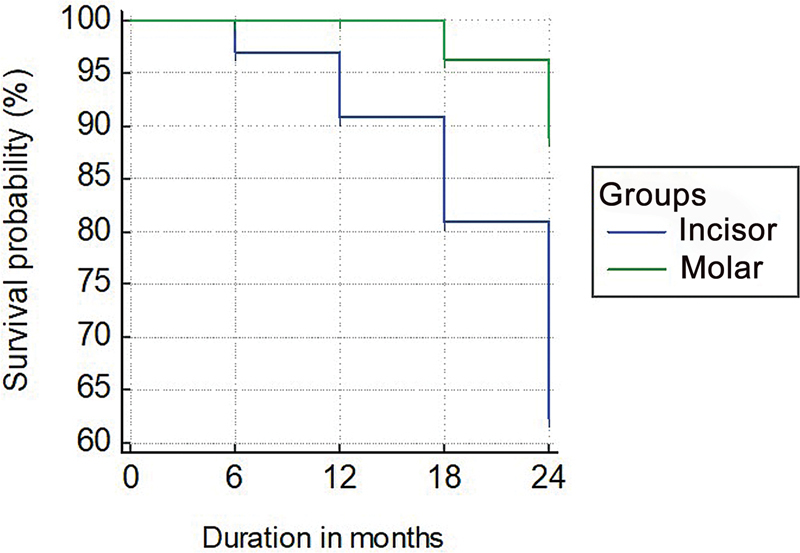
Kaplan–Meier survival graph of incisor and molar group for a period of 2 years.

The prefabricated Zirconia crowns were assessed for restoration failure every 6 months for a 2-year follow-up period ( Table 5 ). The incisors prefabricated Zirconia crowns exhibited an increased incidence of restoration failure than the molar Zirconia crowns. The Kaplan–Meier 2-year survival estimate of the NuSmile Zirconia crowns incisor group was 62.1% (95% CI: 58.5– 65.5), and the molar crowns group was 88% (95% CI: 78–85) survival probability ( Fig. 3 ). Results show that incisor and molar significantly influence the duration of survival time of Zirconia crown, individually.

**Table 5:** Restoration failure and exfoliation of zirconia crowns at 6-, 12-, 18-, and 24 months intervals

Follow up Period	6 ^th^ months		12 ^th^ months		18 ^th^ months		24 ^th^ months	
Type of Crowns	Incisors	Molars	Incisors	Molars	Incisors	Molars	Incisors	Molars
ZR Crowns followed	(n‐ 172)	(n‐ 60)	(n‐ 162)	(n‐ 60)	(n‐ 156)	(n‐ 58)	(n‐ 148)	(n‐ 54)
**Score 0**	153	60	132	58	123	54	114	50
**Score 1**	0	0	0	0	0	0	0	0
**Score 2**	19	0	30	2	30	2	32	4
**Score E**	0	0	0	0	3	2	2	0

Abbreviations: ns, not significant; SD, standard deviation.

Note: Score 0, crown appears normal; Score 1, noticeable area of loss; Score 2, complete loss of crown; Score E, exfoliated, as per the United States Public Health Service criteria.

**Fig. 4:**
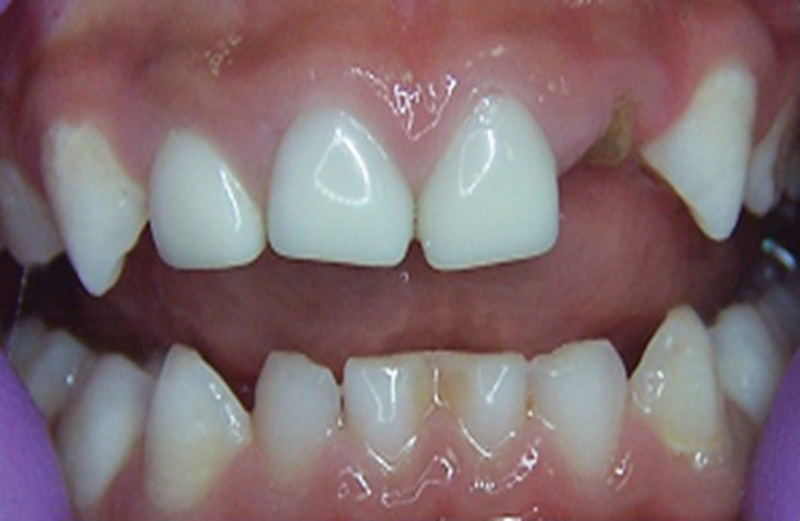
Restoration failure on deciduous lateral incisors.

Out of 172 anterior Zirconia crowns that were followed up at 6 months, 19 crowns were lost completely ( Fig. 4 ). At 24 months review, only 148 incisor crowns were available, out of which 32 teeth had a complete loss of Zirconia crown. The corresponding figures for molar Zirconia crowns were only four crowns out of 54 lost entirely after 2 years.

### Marginal Integrity

**Fig. 5:**
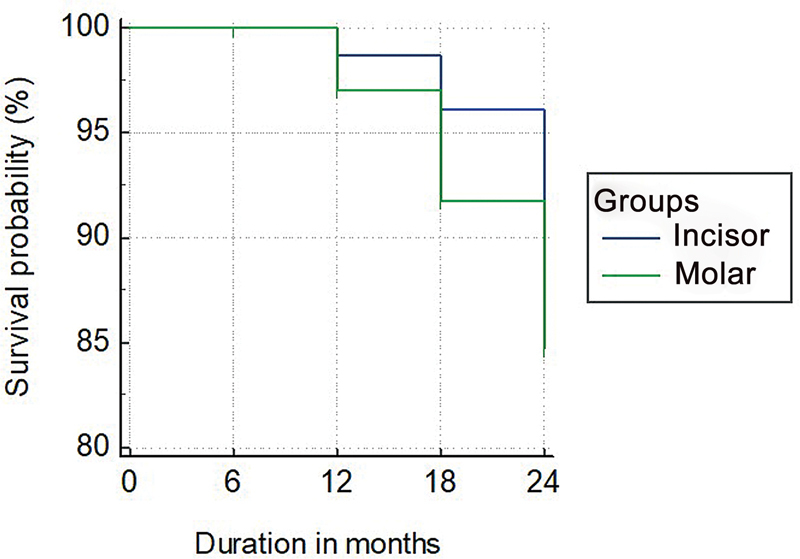
Kaplan–Meier graph for marginal integrity of incisor and molar group for a period of 2 years.

**Fig. 6:**
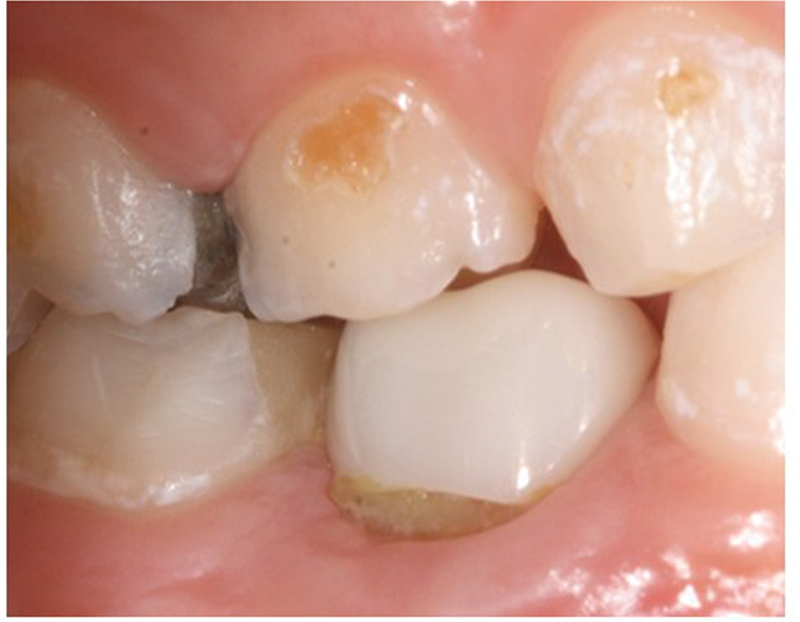
Loss of marginal integrity on lower deciduous first molar when restored with NuSmile Zirconia molar crown.

**Fig. 7:**
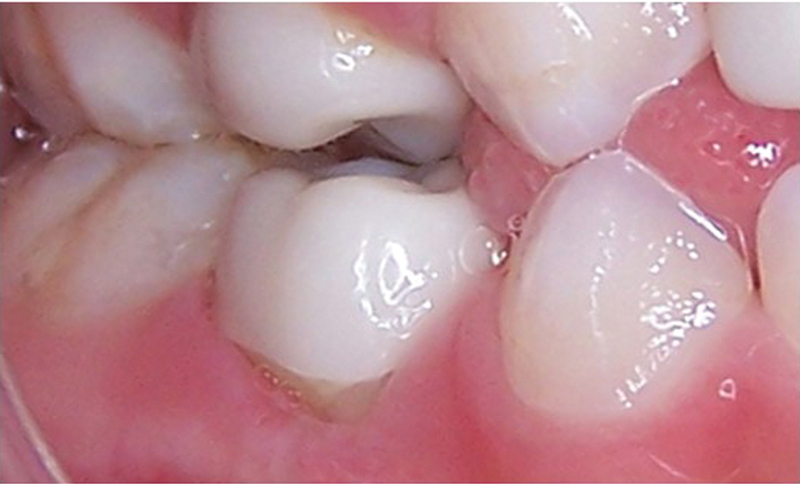
Loss of marginal integrity on lower deciduous first molar for prefabricated Zirconia crown.

**Fig. 8:**
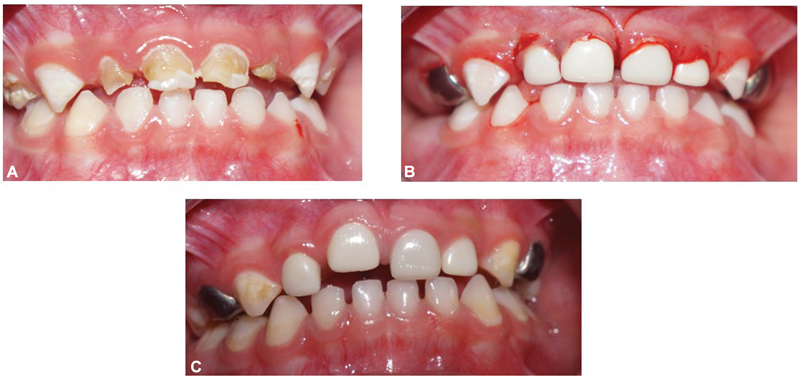
( **A** ) Pre-treatment 51, 52, 61, and 62. ( **B** ) Prefabricated Zirconia crown on 52, 51, 61, and 62. ( **C** ) Prefabricated Zirconia crown on 51 infra-occluded at 12 months.

**Fig. 9:**
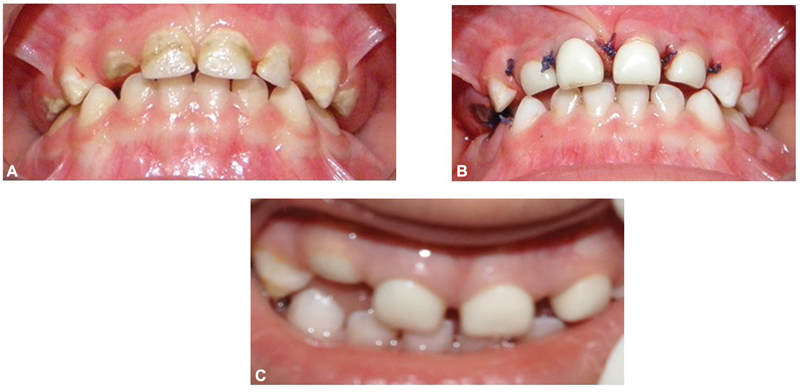
(A) Pre-treatment 51, 52, 61, and 62. ( **B** ) Prefabricated Zirconia crown on 52, 51, 61, and 62. (C) Infra-occluded tooth 52 at 12 months.

**Fig. 10:**
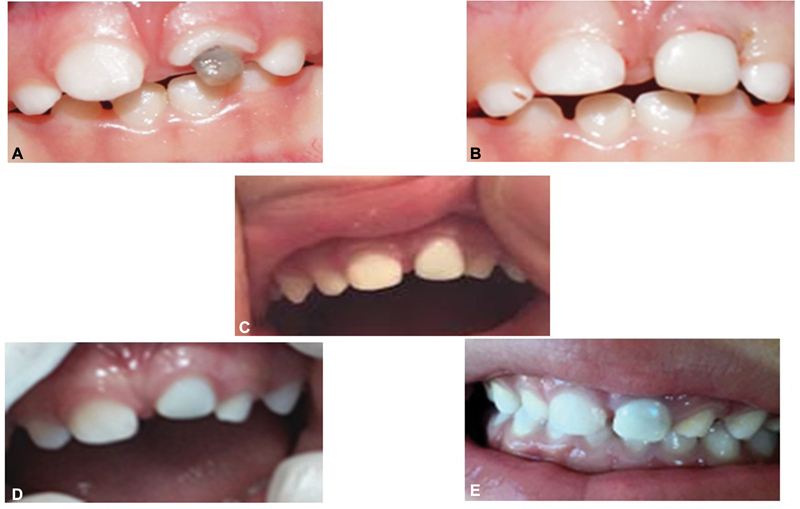
( **A** ) Pre-operative 61 trauma, ( **B** ) Post-operative prefabricated Zirconia crown on 61, ( **C** ) Infra-occluded at 12 months, and ( **D, E** ) Tooth re-eruption.

The 2-year marginal integrity calculated with the Kaplan–Meier method revealed the NuSmile Zirconia crowns incisor group to be 94.5% (95% CI: 92.5–96.5). Kaplan–Meier 2-year marginal integrity estimate of the NuSmile Zirconia crowns incisor group was 92% (95% CI: 96.5–98) and the molar crowns group was 84.5 % (95% CI: 86.5–96) survival probability. The data show that incisor and molar significantly influence the time taken to lose marginal integrity, individually ( Fig. 5 ). Prefabricated Zirconia crowns were checked for their marginal adaptation every 6 months for 2 years ( Table 6 ). The NuSmile Zirconia incisor group had 16 crowns that were infra-occluded and re-erupted within 2 years, and the NuSmile Zirconia molar group had 14 crowns with detectable loss of marginal integrity ( Figs. 6 and 7 ). The results showed that the NuSmile Zirconia incisor crown had greater marginal integrity in the molar group. Figs. 8 , 9 , 10 show various aspects of prefabricated Zirconia crown placement.

**Table 6:** Marginal integrity of zirconia crowns at 6-, 12-, 18-, and 24 months intervals

Follow up Period	6 ^th^ months		12 ^th^ months		18 ^th^ months		24 ^th^ months	
Type of Crowns	Incisors	Molars	Incisors	Molars	Incisors	Molars	Incisors	Molars
ZR Crowns followed	(n‐ 172)	(n‐ 60)	(n‐ 162)	(n‐ 60)	(n‐ 156)	(n‐ 58)	(n‐ 148)	(n‐ 54)
**Score 0**	172	60	155	55	148	53	142	50
**Score 1**	0	0	0	5	0	5	0	4
**Score 2**	0	0	7	0	5	0	4	0
**Score E**	0	0	0	0	3	1	2	0

Abbreviations: ns, not significant; SD, standard deviation.

Note: Score 0, no detectable margin; Score 1, detectable margin; Score 2, infra-occlusion; Score E, exfoliated.

## Discussion

Pediatric dentistry has undergone a paradigm shift from clinical decision-making to a patient-sensitive treatment plan. 12 Childhood caries is a public health problem requiring multisectoral coordination for effective management. 4 13 14

The preformed crown is a promising method of providing substantial shelter for endodontic-treated primary teeth. 4 15 Although an array of prefabricated economic crowns is available in the market, esthetics and the most significant pitfall can be efficiently satisfied by pediatric Zirconia crowns. 16 In this study, NuSmile prefabricated Zirconia crowns were placed more on the anterior teeth than molars. This indicated high esthetic demand currently emerging as an expected standard of care among parents and children, qualifying Zirconia crown as an excellent alternative for other pediatric restorative options. Only a handful of studies have assessed the clinical performance of Zirconia crowns. 1 3 10 17 18 19 20 Moreover, reports of longitudinal evaluations are sparse.

Any metric to be justifiably validated in dentistry needs to be comprehensively drafted to evaluate its survivability, marginal integrity, and effect on surrounding oral structures. Hence, the purpose of this work was to efficiently investigate the longitudinal detailing of NuSmile Zirconia crowns' clinical performance for 2 years. Zirconia pediatric crown requires extensive subgingival preparation; hence, assessing its biocompatibility with gingiva is of paramount importance. In this work, better gingival scores consistently over 2 years were evidenced. This finding was similar to other studies reported earlier. 21 22 23 The average plaque index was higher for the control group than the test group comprising NuSmile prefabricated Zirconia crowns. Decreased plaque score reported in this work was consistent with some previous studies. 11 21 22 23

The biological outcome measured in terms of mean gingival health scores corresponded to the results of plaque level scores for 2 years. This was in line with previous findings that polished and smooth surfaces result in less plaque accumulation and minor gingival irritation. 24 25 Furthermore, the manufacturing method of NuSmile prefabricated Zirconia crown utilizes a proprietary injection molding technique and hand-polishing method, which lowers its lower surface roughness (Ra 2.8) and higher mean gloss (Ga 42.7). 26 The above parameters might have resulted in lesser plaque accumulation and decreased gingival inflammation.

The Zirconia crowns were cemented with conventional glass ionomer cement due to the limited control of hemostasis as the preparation for Zirconia crowns is 1–2 mm subgingival. However, as per manufacturer recommendations, the luting cement of choice for prefabricated zirconium crowns is bioactive resin-based glass ionomer cement which requires good gingival bleeding control prior to cementation. Numerous published studies have highlighted the success of resin-reinforced glass ionomers, 27 28 but a recent study conducted 29 concluded that packable conventional glass ionomer cement is more retentive than bioactive cement for the cementation of primary Zirconia crowns.

The survival rate measured in terms of restoration failure for NuSmile prefabricated Zirconia restoration in this work reported no chipping or fracturing. Only loss of crowns was evaluated at each follow-up visit. The endodontic failures were not evaluated and, hence, not included. The absence of porcelain-veneered restoration and monolithic Zirconia preparation, which was said to exhibit higher resistance to fracture loads than layered Zirconia crowns and metal-ceramic crowns. 30 31 The restoration failure in terms of complete loss of crown was higher for incisor crowns than for the molar prefabricated Zirconia group. This may be due to increased surface area and anatomy of posterior teeth compared with its anterior counterpart that offer better retention. More overstudies reported that it was comparatively convenient to fit Zirconia crown in molars. 15 In all cases, restoration failure happened at the crown-to-teeth interface and not on the cement-to-crown interface as no luting cement remained on the tooth structure after the debonding of Zirconia crowns.

Marginal integrity is an integral component of clinical performance. It has many subsets of factors—the first being marginal fit. The excellent finish line of the study group could have contributed to a high marginal fit in this study. The second factor is the vertical gap between the restoration and the prepared tooth abutment. Creating a favorable area for bacterial accumulate in the marginal gap jeopardizes the longevity of the restoration, leading to the development of secondary caries. No evidence of secondary caries in this study substantiates good marginal fit and a minimal vertical gap in this pediatric crown.

In the present study, traumatized teeth that were infra-occluded and restored with the Zirconia crowns re-erupted along with the natural tooth, spontaneously suggesting a favorable prognosis. 32 However, periodic clinical and radiographic follow-up should be performed to prevent pulp infection of the intruded teeth and possible disturbances to the budding permanent tooth.

The strength of this research work is a sufficient sample size and comprehensive clinical performance evaluation longitudinally. Limitations of the present study were that both patients under general and local anesthesia formed the study cohort that could have resulted in mixed clinical outcomes challenging the external validity. Since it is a retrospective study, potential confounding factors such as patient selection, operator bias, and lack of standardization between operators can have an effect on the results. Moreover, further long-term studies among different commercially available Zirconia crowns can help us understand the differences in survival and biological outcomes in each group and foster amendments as needed. To summarize, pediatric Zirconia crowns have demonstrated better gingival health, decreased plaque accumulation, and reasonable survival rates longitudinally, especially for molars. Further research on various brands of Zirconia crowns and methods for longer retention of crowns in anterior teeth should be planned.

## Conclusion

Based on the findings of this study, we conclude that Zirconia pediatric crowns preserve and maintain gingival health. They also prevent microbial plaque accumulation similar to the natural tooth. These pediatric crowns have long-term survival rates with good retention and marginal integrity, indirectly preventing secondary caries. Furthermore, these crowns do not intervene with the path of eruption of infra-occluded traumatized teeth. Hence, Zirconia pediatric crowns are a well-suited restoration of choice for primary teeth rehabilitation.

## References

[1] Walia T Salami A A Bashiri R Hamoodi O M Rashid F A randomised controlled trial of three aesthetic full-coronal restorations in primary maxillary teeth Eur J Paediatr Dent 2014 15 02 113 118 25102458

[2] Pani S C Saffan A A AlHobail S Bin Salem F AlFuraih A AlTamimi M Esthetic concerns and acceptability of treatment modalities in primary teeth: a comparison between children and their parents Int J Dent 2016 2016 3.163904E6 10.1155/2016/3163904 PMC4942659 27446212

[3] Aiem E Smaïl-Faugeron V Muller-Bolla M Aesthetic preformed paediatric crowns: systematic review Int J Paediatr Dent 2017 27 04 273 282 27532506 10.1111/ipd.12260

[4] Innes N P Ricketts D Chong L Y Keightley A J Lamont T Santamaria R M Preformed crowns for decayed primary molar teeth Cochrane Database Syst Rev 2015 2015 12 CD005512 26718872 10.1002/14651858.CD005512.pub3 PMC7387869

[5] Bell S J Morgan A G Marshman Z Rodd H D Child and parental acceptance of preformed metal crowns Eur Arch Paediatr Dent 2010 11 05 218 224 20932394 10.1007/BF03262750

[6] Al Shobber M Z Alkhadra T A Fracture resistance of different primary anterior esthetic crowns Saudi Dent J 2017 29 04 179 184 29033529 10.1016/j.sdentj.2017.07.006 PMC5634802

[7] Larsson C Zirconium dioxide based dental restorations. Studies on clinical performance and fracture behaviour Swed Dent J Suppl 2011 213 9 84 21919311

[8] El Shahawy O I O'Connell A C Successful restoration of severely mutilated primary incisors using a novel method to retain zirconia crowns—two year results J Clin Pediatr Dent 2016 40 06 425 430 27805887 10.17796/1053-4628-40.6.425

[9] Bolaca A Erdogan Y *In vitro* evaluation of the wear of primary tooth enamel against different ceramic and composite resin materials Niger J Clin Pract 2019 22 03 313 319 30837417 10.4103/njcp.njcp_358_18

[10] Holsinger D M Wells M H Scarbecz M Donaldson M Clinical evaluation and parental satisfaction with pediatric zirconia anterior crowns Pediatr Dent 2016 38 03 192 197 27306242

[11] American Academy of Pediatric Dentistry Pulp therapy for primary and immature permanent teeth The Reference Manual of Pediatric Dentistry 2020 399 407

[12] Mathew M G Roopa K B Soni A J Khan M M Kauser A Evaluation of clinical success, parental and child satisfaction of stainless steel crowns and zirconia crowns in primary molars J Family Med Prim Care 2020 9 03 1418 1423 32509626 10.4103/jfmpc.jfmpc_1006_19 PMC7266243

[13] Meng X Zhang J Chen J KR-12 coating of polyetheretherketone (PEEK) surface *via* polydopamine improves osteointegration and antibacterial activity *in vivo* J Mater Chem B Mater Biol Med 2020 8 44 10190 10204 33103697 10.1039/d0tb01899f

[14] Teng R Meng Y Zhao X Combination of polydopamine coating and plasma pretreatment to improve bond ability between PEEK and primary teeth Front Bioeng Biotechnol 2021 8 630094 33585424 10.3389/fbioe.2020.630094 PMC7880054

[15] Lopez Cazaux S Hyon I Prud'homme T Dajean Trutaud S Twenty-nine-month follow-up of a paediatric zirconia dental crown BMJ Case Rep 2017 2017 bcr-2017-219891 10.1136/bcr-2017-219891 PMC5534797 28619974

[16] Ajayakumar L P Chowdhary N Reddy V R Chowdhary R Use of restorative full crowns made with zirconia in children: a systematic review Int J Clin Pediatr Dent 2020 13 05 551 558 33623346 10.5005/jp-journals-10005-1822 PMC7887175

[17] Ashima G Sarabjot K B Gauba K Mittal H C Zirconia crowns for rehabilitation of decayed primary incisors: an esthetic alternative J Clin Pediatr Dent 2014 39 01 18 22 25631720 10.17796/jcpd.39.1.t6725r5566u4330g

[18] Planells del Pozo P Fuks A B Zirconia crowns—an esthetic and resistant restorative alternative for ECC affected primary teeth J Clin Pediatr Dent 2014 38 03 193 195 25095311 10.17796/jcpd.38.3.0255q84jt2851311

[19] Salami A Walia T Bashiri R Comparison of parental satisfaction with three tooth-colored full-coronal restorations in primary maxillary incisors J Clin Pediatr Dent 2015 39 05 423 428 26551364 10.17796/1053-4628-39.5.423

[20] Seminario A L Garcia M Spiekerman C Rajanbabu P Donly K J Harbert P Survival of zirconia crowns in primary maxillary incisors at 12-, 24- and 36-month follow-up Pediatr Dent 2019 41 05 385 390 31648670

[21] Abdulhadi B Abdullah M Alaki S Alamoudi N Attar M Clinical evaluation between zirconia crowns and stainless steel crowns in primary molars teeth J Paediatr Dent 2017 5 01 21

[22] Hanafi L Altinawi M Comisi J C Evaluation and comparison two types of prefabricated zirconia crowns in mixed and primary dentition: a randomized clinical trial Heliyon 2021 7 02 e06240 33665422 10.1016/j.heliyon.2021.e06240 PMC7900688

[23] Taran P K Kaya M S A comparison of periodontal health in primary molars restored with prefabricated stainless steel and zirconia crowns Pediatr Dent 2018 40 05 334 339 30355428

[24] Song F Koo H Ren D Effects of material properties on bacterial adhesion and biofilm formation J Dent Res 2015 94 08 1027 1034 26001706 10.1177/0022034515587690

[25] Teughels W Van Assche N Sliepen I Quirynen M Effect of material characteristics and/or surface topography on biofilm development Clin Oral Implants Res 2006 17 02 68 81 16968383 10.1111/j.1600-0501.2006.01353.x

[26] Theriot A L Frey G N Ontiveros J C Badger G Gloss and surface roughness of anterior pediatric zirconia crowns J Dent Child (Chic) 2017 84 03 115 119 29282166

[27] Donly K J Sasa I Contreras C I Mendez M JC Prospective randomized clinical trial of primary molar crowns: 24-month results Pediatr Dent 2018 40 04 253 258 30345963

[28] Talekar A L Chaudhari G S Waggoner W F Chunawalla Y K An 18-month prospective randomized clinical trial comparing zirconia crowns with glass-reinforced fiber composite crowns in primary molar teeth Pediatr Dent 2021 43 05 355 362 34654496

[29] Azab M M Moheb D M El Shahawy O I Rashed M A-M Influence of luting cement on the clinical outcomes of zirconia pediatric crowns: a 3-year split-mouth randomized controlled trial Int J Paediatr Dent 2020 30 03 314 322 31845435 10.1111/ipd.12607

[30] Beuer F Stimmelmayr M Gueth J F Edelhoff D Naumann M In vitro performance of full-contour zirconia single crowns Dent Mater 2012 28 04 449 456 22196898 10.1016/j.dental.2011.11.024

[31] Sun T Zhou S Lai R Load-bearing capacity and the recommended thickness of dental monolithic zirconia single crowns J Mech Behav Biomed Mater 2014 35 93 101 24762856 10.1016/j.jmbbm.2014.03.014

[32] Hirata R Kaihara Y Suzuki J Kozai K Management of intruded primary teeth after traumatic injuries Pediatr Dent J 2011 21 02 94 100

